# Supplementary material for: Associations between relative grip strength and type 2 diabetes mellitus: The Yangpyeong cohort of the Korean genome and epidemiology study
Source: PLoS One. 2021 Aug 26;16(8):e0256550. doi: 10.1371/journal.pone.0256550 (PMC8389482; doi:10.1371/journal.pone.0256550)
Supplement: S1 Table — (DOCX) [file pone.0256550.s001.docx]

| **S1 Table. Odds ratios of T2DM by tertiles of relative grip strength.** | | | | | |
| --- | --- | --- | --- | --- | --- |
| **Relative grip strength** | **N** | **Cases** | **OR (95% CI)** | | |
|  |  |  | **Model 1** | **Model 2** | **Model 3** |
| **By tertiles** |  |  |  |  |  |
| Q1 (weakest) | 937 | 161 | 1.00 (reference) | 1.00 (reference) | 1.00 (reference) |
| Q2 | 937 | 120 | **0.74 (0.57-0.96)** | **0.73 (0.56-0.96)** | 0.77 (0.59-1.01) |
| Q3 (strongest) | 937 | 90 | **0.56 (0.42-0.75)** | **0.60 (0.45-0.81)** | **0.69 (0.50-0.94)** |
| P for linear trend |  |  | **<0.001** | **<0.001** | **0.016** |
| **By quartiles** |  |  |  |  |  |
| Q1 (weakest) | 703 | 129 | 1.00 (reference) | 1.00 (reference) | 1.00 (reference) |
| Q2 | 703 | 100 | 0.76 (0.57-1.02) | **0.72 (0.53-0.97)** | 0.75 (0.55-1.00) |
| Q3 | 703 | 81 | **0.62 (0.45-0.84)** | **0.62 (0.46-0.86)** | **0.68 (0.49-0.94)** |
| Q4 (strongest) | 702 | 61 | **0.46 (0.33-0.65)** | **0.51 (0.36-0.71)** | **0.58 (0.40-0.83)** |
| P for linear trend |  |  | **<0.001** | **<0.001** | **0.002** |
| Model 1 was adjusted for sex and age (years).  Model 2 was adjusted for Model 1 plus smoking status (never, former, current), current alcohol drinking status (yes or no), regular exercise (yes or no), living with family (yes or no), ≥high school graduate (yes or no), family history of diabetes (yes or no), hypertension (yes or no), and dyslipidemia (yes or no). Model 3 was adjusted for Model 2 plus body mass index (kg/m^2^) | | | | | |
