# Supplementary material for: Associations between relative grip strength and type 2 diabetes mellitus: The Yangpyeong cohort of the Korean genome and epidemiology study
Source: PLoS One. 2021 Aug 26;16(8):e0256550. doi: 10.1371/journal.pone.0256550 (PMC8389482; doi:10.1371/journal.pone.0256550)
Supplement: S2 Table — (DOCX) [file pone.0256550.s002.docx]

| **S2 Table. Odds ratios of T2DM by quintiles of grip strength divided by body mass index.** | | | | | |
| --- | --- | --- | --- | --- | --- |
| **Relative grip strength** | **N** | **Cases** | **OR (95% CI)** | | |
|  |  |  | **Model 1** | **Model 2** | **Model 3** |
| Q1 (weakest) | 563 | 107 | 1.00 (reference) | 1.00 (reference) | 1.00 (reference) |
| Q2 | 562 | 81 | 0.91 (0.66-1.25) | 0.88 (0.63-1.22) | 0.92 (0.66-1.28) |
| Q3 | 562 | 70 | **0.76 (0.54-1.07)** | 0.74 (0.52-1.04) | 0.80 (0.56-1.14) |
| Q4 | 562 | 72 | **0.62 (0.43-0.89)** | **0.62 (0.43-0.90)** | 0.71 (0.48-1.04) |
| Q5 (strongest) | 562 | 41 | **0.57 (0.39-0.84)** | **0.63 (0.43-0.94)** | 0.76 (0.50-1.17) |
| P for linear trend |  |  | **<0.001** | **0.004** | 0.081 |
| Per SD in relative grip strength |  |  | **0.71 (0.59-0.86)** | **0.73 (0.60-0.88)** | **0.79 (0.65-0.97)** |
| Model 1 was adjusted for sex and age (years).  Model 2 was adjusted for Model 1 plus smoking status (never, former, current), current alcohol drinking status (yes or no), regular exercise (yes or no), living with family (yes or no), ≥high school graduate (yes or no), family history of diabetes (yes or no), hypertension (yes or no), and dyslipidemia (yes or no). Model 3 was adjusted for Model 2 plus body mass index (kg/m^2^). | | | | | |
