# Supplementary material for: Associations between relative grip strength and type 2 diabetes mellitus: The Yangpyeong cohort of the Korean genome and epidemiology study
Source: PLoS One. 2021 Aug 26;16(8):e0256550. doi: 10.1371/journal.pone.0256550 (PMC8389482; doi:10.1371/journal.pone.0256550)
Supplement: S3 Table — (DOCX) [file pone.0256550.s003.docx]

| **S3 Table. Odds ratio of prevalence of type 2 diabetes mellitus, stratified by relative grip strength in subgroup.** | | | | | | | | | |
| --- | --- | --- | --- | --- | --- | --- | --- | --- | --- |
|  |  | | **Quintiles of relative grip strength, OR (95% CI)^a^** | | | | |  |  |
| **All** | **N** | **Cases** | **Q1 (weakest)** | **Q2** | **Q3** | **Q4** | **Q5 (strongest)** | **P-trend** |  |
| **BMI** |  |  |  |  |  |  |  |  |  |
| Normal weight | 910 | 80 | 1.00 (reference) | 0.91 (0.38-2.18) | 0.42 (0.15-1.17) | 0.64 (0.28-1.49) | 0.54 (0.24-1.24) | 0.116 |  |
| Overweight | 715 | 64 | 1.00 (reference) | 0.76 (0.33-1.76) | 0.92 (0.42-2.04) | 0.79 (0.34-1.82) | 0.43 (0.15-1.25) | 0.213 |  |
| Obese | 1167 | 168 | 1.00 (reference) | **0.61 (0.38-0.97)** | 0.72 (0.45-1.17) | 0.71 (0.41-1.23) | **0.27 (0.09-0.80)** | **0.036** |  |
| **Living with family** |  |  |  |  |  |  |  |  |  |
| Yes | 2474 | 287 | 1.00 (reference) | 0.71 (0.49-1.04) | 0.71 (0.48-1.05) | 0.71 (0.47-1.07) | **0.49 (0.30-0.81)** | **0.014** |  |
| No | 318 | 25 | 1.00 (reference) | 0.34 (0.08-1.40) | 0.52 (0.16-1.66) | 0.47 (0.12-1.81) | 0.31 (0.05-1.80) | 0.203 |  |
| **Education** |  |  |  |  |  |  |  |  |  |
| > high school diploma | 879 | 83 | 1.00 (reference) | 0.52 (0.24-1.14) | 0.61 (0.29-1.29) | 0.59 (0.27-1.28) | **0.36 (0.15-0.91)** | 0.087 |  |
| ≤ high school diploma | 879 | 83 | 1.00 (reference) | 0.76 (0.51-1.15) | 0.72 (0.46-1.11) | 0.72 (0.45-1.14) | **0.51 (0.29-0.89)** | **0.026** |  |
| **Family history of T2DM** |  |  |  |  |  |  |  |  |  |
| Yes | 869 | 139 | 1.00 (reference) | 0.76 (0.36-1.61) | 1.35 (0.64-2.83) | 1.54 (0.73-3.23) | 0.94 (0.37-2.42) | 0.334 |  |
| No | 1923 | 173 | 1.00 (reference) | 0.68 (0.45-1.03) | **0.54 (0.35-0.84)** | **0.47 (0.29-0.77)** | **0.37 (0.21-0.64)** | **<0.001** |  |
| **Hypertension** |  |  |  |  |  |  |  |  |  |
| Yes | 869 | 139 | 1.00 (reference) | 0.82 (0.47-1.42) | 0.85 (0.48-1.49) | 0.76 (0.42-1.39) | 0.54 (0.25-1.17) | 0.158 |  |
| No | 1923 | 173 | 1.00 (reference) | **0.61 (0.38-0.99)** | 0.62 (0.38-1.02) | 0.63 (0.37-1.06) | **0.45 (0.25-0.83)** | **0.022** |  |
| Data presented as adjusted odds ratio (95% confidence interval). ORs are presented per quintile increase in relative grip strength strata.  Analyses were adjusted for sex and age (years), smoking status (never, former, current), current alcohol drinking status (yes or no), regular exercise (yes or no), living with family (yes or no, not in living with family-stratified analyses), ≥high school graduate (yes or no, not in ≥high school graduate-stratified analyses), family history of diabetes (yes or no, not in family history of diabetes-stratified analyses), hypertension (yes or no, not in hypertension-stratified analyses), dyslipidemia (yes or no, not in dyslipidemia-stratified analyses), and BMI (kg/m^2^, not in body mass index-stratified analyses). Normal weight was BMI < 23.0 kg/m^2^, overweight was 23.0-24.9c kg/m^2^, and obese was ≥25.0 kg/m^2^. No subgroup analyses were conducted for dyslipidemia. Because of small sample size that was responded for yes (n=65; 2.3% of sample) and limited number of T2DM cases in this group. (n=20) | | | | | | | | | |
