# Supplementary material for: Associations between relative grip strength and type 2 diabetes mellitus: The Yangpyeong cohort of the Korean genome and epidemiology study
Source: PLoS One. 2021 Aug 26;16(8):e0256550. doi: 10.1371/journal.pone.0256550 (PMC8389482; doi:10.1371/journal.pone.0256550)
Supplement: Attachment — Submitted filename: Response to Reviewers Comments.docx [file pone.0256550.s004.docx]

**Associations between Relative Grip Strength and Type 2 Diabetes Mellitus:**

**The Yangpyeong Cohort of the Korean Genome and Epidemiology Study**

We thank the editor and reviewers for their helpful edits and comments, which have improved the manuscript, and we are grateful for the opportunity to provide a revised version for consideration. We have carefully considered, discussed, and responded to each comment below, with direct references to the locations (page and line numbers) of changes in the revised manuscript by using track changes.

**■ Responses to Editors comments**

| **Comments** | **Author’s response** |
| --- | --- |
| 1) When submitting your revision, we need you to address these additional requirements. Please ensure that your manuscript meets PLOS ONE's style requirements, including those for file naming. | According to PLOS ONE’S style requirement, we have revised the formatting throughout the manuscript, and we have submitted our manuscript with the following three file names:  ‘Response to Reviewers’. ‘Revised Manuscript with Track Changes’, ‘Manuscript’. |
| 2) In ethics statement in the manuscript and in the online submission form, please provide additional information about the patient records/samples used in your retrospective study. Specifically, please ensure that you have discussed whether all data/samples were fully anonymized before you accessed them and/or whether the IRB or ethics committee waived the requirement for informed consent. If patients provided informed written consent to have data/samples from their medical records used in research, please include this information. | We have corrected this issue in the revised manuscript (Page 5, Lines 74-80) and online submission form, with the following sentences:  “The study was approved by the Institutional Review Board of Hanyang University (IRB No: HYUN IRB 2005-15). The study was conducted in accordance with the Declaration of Helsinki. All patients provided written informed consent, and all data were fully anonymized prior to analysis.” |

| **Comments** | **Author’s response** |
| --- | --- |
| 3) Please provide an amended statement that declares *all* the funding or sources of support (whether external or internal to your organization) received during this study, as detailed online in our guide for authors at http://journals.plos.org/plosone/s/submit-now. Please also include the statement “There was no additional external funding received for this study.” in your updated Funding Statement.  Please include your amended Funding Statement within your cover letter. We will change the online submission form on your behalf. | Following the journal’s guideline, we added the information of the author who received the grant. Our new funding statement is included in our cover letter and below to be corrected in the online submission form:  “This work was partly supported by the Research Program funded by the Korean Centers for Disease Control and Prevention (grant numbers: 2004-E71004-00, 2005-E71011-00, 2006-E71009-00, 2007-E71002-00, 2008-E71004-00, 2009-E71006-00, 2010-E71003-00, 2011-E71002-00, 2012-E71007-00, 2013-E71008-00, 2014-E71006-00, 2014-E71006-01, 2016-E71001-00, grant receiver: BY Choi). There was no additional external funding received for this study. The funders had no role in study design, data collection, analysis, decision to publish, or preparation of the manuscript.” |
| 4) In your revised cover letter, please address the following prompts:  4a) If there are ethical or legal restrictions on sharing a de-identified data set, please explain them in detail (e.g., data contain potentially identifying or sensitive patient information) and who has imposed them (e.g., an ethics committee). Please also provide contact information for a data access committee, ethics committee, or other institutional body to which data requests may be sent.  4b) If there are no restrictions, please upload the minimal anonymized data set necessary to replicate your study findings as either Supporting Information files or to a stable, public repository and provide us with the relevant URLs, DOIs, or accession numbers. Please see http://www.bmj.com/content/340/bmj.c181.long for guidelines on how to de-identify and prepare clinical data for publication. For a list of acceptable repositories, please see http://journals.plos.org/plosone/s/data-availability#loc-recommended-repositories. | 4a) We have corrected this in the cover letter as demonstrated below:  “The data used in our study were limited to research purposes only and cannot be made publicly available due to privacy policy. Data are owned and distributed by The Korea Centers for Disease Control and Prevention (KCDC). The data are available from the Division of Epidemiology and Health Index, KCDC (http://cdc.go.kr, Tel; 82-43-719-6745).”  4b) Due to the restrictions imposed by the KCDC, we are unable to upload the minimal anonymized data. |

**■ Responses to Reviewer #1’s comments**

| **Comments** | **Author’s response** |
| --- | --- |
| **General comments**  This is a cross sectional study exploring the relation of relative grip strength and type 2 diabetes risk in a Korean population. The study is straightforward although the Introduction and Discussion could be enhanced for better readability. I have some concerns over the statistical analyses and the way how the exposures were being categorized in the analyses. Please see below for my comments. | Thank you for the overview – we hope to have addressed your concerns below. |
| **Major comments 1)**  The Introduction should be revised to capture the key points the study is trying to address. I think the issue with previous studies is the lack of consideration of body mass index which is related to grip strength. It would be helpful for authors to explain why considering relative grip strength and BMI altogether with type 2 diabetes is helpful, especially when relative grip strength takes into account the effect of BMI in the calculation. | We modified our introduction to emphasize the importance of examining the relationship between relative grip strength and BMI for T2DM (page 4-5, lines 44-62) and included here:  “Studies have shown that relative grip strength, which is grip strength divided by body weight or BMI, may be more strongly associated with metabolic syndrome and cardiometabolic risk factors (e.g., blood pressure, triglyceride, fasting glucose) than absolute grip strength. Thus, relative grip strength may have a stronger association with T2DM than absolute grip strength.  Obesity is attributed to insulin resistance by permanently increasing free fatty acids in plasma and reducing glucose uptake by muscle. Many previous studies have elucidated the importance of obesity, assessed by body mass index (BMI) ≥30 kg/m^2^, as a prominent risk factor for T2DM. Thus, the association between grip strength and T2DM may depend on BMI status. Previous studies investigating the relationship between grip strength and T2DM have controlled for BMI [18], however, few have investigated how the association between relative grip strength and T2DM is varied with BMI status.  It is important to examine the combined effects of muscular strength and overweight/obesity since both are important to develop public health recommendations and policies. For example, if people with overweight/obesity can reduce the risk of T2DM by improving muscular strength, this has a large clinical and public health impact since overweight/obesity is prevalent in most mid- and high-income countries [19]. Further, there is still limited data on the relative contributions of muscular strength versus overweight/obesity to T2DM.” |

| **Comments** | **Author’s response** |
| --- | --- |
| **Major comments 2)**  Have the authors considered using other body composition metrics as the BMI to calculate relative grip strength? If the authors preferred to use body weight, please also provide justifications (PMID: 20191251; 27559733). This would also have implications for all downstream analyses. | Thank you for the great comment. In several previous studies, grip strength normalized to body weight was significantly associated with type 2 diabetes (Karvonen-Gutierrez et al, *Age and ageing*, 2018; Momma et al, *Journal of epidemiology*, 2019; Ntuk et al, *Diabetic Medicine*, 2017; 30058613; 28144980]. In addition, Chun et al. (Chun et al, *PloS one*, 2019) point out that grip strength normalized to body weight was more strongly associated with metabolic syndrome than grip strength normalized to BMI. Given these previous investigations in type 2 diabetes and metabolic syndrome, we have also chosen to use grip strength normalized to body weight for easier comparison with prior literature. Additionally, following the comment, we have performed and included a sensitivity analysis using relative grip strength normalized to BMI as Table 2 in our Supplementary Materials as present below. The association was attenuated when we use BMI instead of body weight in Model 1 and 2. Moreover, ORs were no longer significant in Model 3. **Supplementary Table 2. Odd Ratios of T2DM by quintiles of relative grip strength divided by body mass index.** \| \| \| \| \|; **Relative grip strength** \| **N** \| **Cases** \| **OR (95% CI)** \| \|; \| \| \| **Model 1** \| **Model 2** \| **Model 3**; Q1 (weakest) \| 563 \| 107 \| 1.00 (reference) \| 1.00 (reference) \| 1.00 (reference); Q2 \| 562 \| 81 \| 0.91 (0.66-1.25) \| 0.88 (0.63-1.22) \| 0.92 (0.66-1.28); Q3 \| 562 \| 70 \| **0.76 (0.54-1.07)** \| 0.74 (0.52-1.04) \| 0.80 (0.56-1.14); Q4 \| 562 \| 72 \| **0.62 (0.43-0.89)** \| **0.62 (0.43-0.90)** \| 0.71 (0.48-1.04); Q5 (strongest) \| 562 \| 41 \| **0.57 (0.39-0.84)** \| **0.63 (0.43-0.94)** \| 0.76 (0.50-1.17); P for linear trend \| \| \| **<.001** \| **0.004** \| 0.081; Per SD in relative grip strength \| \| \| **0.71 (0.59-0.86)** \| **0.73 (0.60-0.88)** \| **0.79 (0.65-0.97)**; Model 1 was adjusted for sex and age (years). Model 2 was adjusted for Model 1 plus smoking status (never, former, current), current alcohol drinking status (yes or no), regular exercise (yes or no), living with family (yes or no), ≥high school graduate (yes or no), family history of diabetes (yes or no), hypertension (yes or no), dyslipidemia (yes or no). Model 3 was adjusted for Model 2 plus body mass index (kg/m^2^). \| \| \| \| \| - Karvonen-Gutierrez CA, Peng Q, Peterson M. Low grip strength predicts incident diabetes among mid-life women: the Michigan Study of Women’s Health Across the Nation. *Age and ageing*, 2018;47(5):685-691. - Momma H, Sawada SS, Kato K. Physical fitness tests and type 2 diabetes among Japanese: a longitudinal study from the Niigata Wellness Study. *Journal of epidemiology*, 2019;29(4):139-146. - Ntuk UE, Celis‐Morales CA, Mackay DF. Association between grip strength and diabetes prevalence in black, South‐Asian, and white European ethnic groups: a cross‐sectional analysis of 418 656 participants in the UK Biobank study. *Diabetic Medicine*, 2017;34(8):1120-1128. - Chun SW, Kim W, Choi KH. Comparison between grip strength and grip strength divided by body weight in their relationship with metabolic syndrome and quality of life in the elderly. *PloS one*, 2019;14(9):e0222040. |
| **Major comments 3)**  It would be good for the authors to repeat the analyses using the actual values of the relative grip strength to assess robustness of findings by different ways of categorization. This will maximize the use of data rather than categorizing them into tertiles or quartiles. | We appreciate the reviewer’s comment and feel this analysis substantially adds to our findings. We have included the results of using relative grip strength as a continuous value in the revised manuscript (Table 2). **Table 2. Odd Ratios of T2DM by quintiles of relative grip strength.** \| \| \| \| \|; **Relative grip strength** \| **N** \| **Cases** \| **OR (95% CI)** \| \|; \| \| \| **Model 1** \| **Model 2** \| **Model 3**; Q1 (weakest) \| 563 \| 107 \| 1.00 (reference) \| 1.00 (reference) \| 1.00 (reference); Q2 \| 562 \| 81 \| 0.75 (0.54-1.02) \| **0.71 (0.51-0.99)** \| 0.73 (0.53-1.02); Q3 \| 562 \| 70 \| **0.64 (0.46-0.89)** \| **0.65 (0.46-0.91)** \| **0.68 (0.48-0.97)**; Q4 \| 562 \| 72 \| **0.68 (0.48-0.95)** \| **0.66 (0.46-0.93)** \| 0.72 (0.50-1.03); Q5 (strongest) \| 562 \| 41 \| **0.37 (0.25-0.55)** \| **0.42 (0.28-0.63)** \| **0.48 (0.32-0.74)**; P for linear trend \| \| \| **<.001** \| **<.001** \| **0.002**; Per SD in relative grip strength^a^ \| \| \| **0.69 (0.59-0.81)** \| **0.72 (0.61-0.84)** \| **0.76 (0.64-0.91)**; ^a^A 1 SD in relative grip strength is equivalent to 0.11kg. Model 1 was adjusted for sex and age (years). Model 2 was adjusted for Model 1 plus smoking status (never, former, current), current alcohol drinking status (yes or no), regular exercise (yes or no), living with family (yes or no), ≥high school graduate (yes or no), family history of diabetes (yes or no), hypertension (yes or no), dyslipidemia (yes or no). Model 3 was adjusted for Model 2 plus body mass index (kg/m^2^). \| \| \| \| \| And have added the following sentences to our Materials and Methods section and Results section: 1) Materials and Methods section: “In addition, the ORs for the prevalence of T2DM per SD change were also calculated.” (page 9, line 152-153). 2) Results section: “Moreover, the OR for T2DM per SD increase in relative grip strength was 0.76 (0.64-0.91), after adjustment for potential confounders in model 3.” (page 12, line 200-201). We have also made our sensitivity analysis for the categorization into tertiles and quartiles available to readers as Table 1, respectively, in the Supplementary Materials. |

| **Comments** | **Author’s response** |
| --- | --- |
| **Major comments 4)**  It would be more preferable to assess potential interactions using formal interaction analyses such as the significance of the interaction term. The low number of diabetes case may also make particular subgroup analyses lack sufficient statistical power. The way how grip strength is being dichotomized appears arbitrary (20%) and require further justification. | We thank the reviewer for their suggestion and feel this additional analysis strengthens our findings looking at the combined effects of relative handgrip strength and BMI. We additionally examined the interaction in our subgroup analyses and updated Fig 1. We did not observe any significant interaction effects for sex, age, smoking, alcohol drinking, or regular exercise. This has been added to our results section (page 12, line 199-200) as follows:  “Associations between relative grip strength and the odds of T2DM revealed consistent trends across all strata with no significant interaction effects observed (p>0.05)”;  In regard to our categorization of grip strength into the categories weak (Lower 20%) or strong (Higher 80%), we have added the following sentence in Materials and methods section in the revised manuscript (page 9-10, line 168-174).  “This categorization was based on the recommendation of the Asian Working Group for Sarcopenia (AWGS) (Chen et al, *Journal of the American Medical Directors Association*, 2014). AWGS recommended using the lower 20th percentile of grip strength of the study population as the cutoff value for low muscle strength before if outcome-based data is unavailable. Although AWGS suggested being defined low grip strength as <26 kg for men and <18kg for women, we cannot use this absolute cutoff value because we used relative grip strength. The method using the cutoff at the 20th percentile was reported in previous studies (Yoo et al, *Journal of Korean medical science*, 2017; Paek et al, *BMJ open,* 2019; Prasitsiriphon et al, *Clinical Medicine Insights: Cardiology*, 2018).”   - Chen LK, Liu LK., Woo J. Sarcopenia in Asia: consensus report of the Asian Working Group for Sarcopenia. *Journal of the American Medical Directors Association*, 2014;*15*(2):95-101. - Yoo JI, Choi H, Ha YC Mean hand grip strength and cut-off value for sarcopenia in Korean adults using KNHANES VI. *Journal of Korean medical science*, 2017;*32*(5):868. - Paek J, Choi YJ. Association between hand grip strength and impaired health-related quality of life in Korean cancer survivors: a cross-sectional study. *BMJ open*, 2019;*9*(9):e030938. - Prasitsiriphon O, Pothisiri W. Associations of grip strength and change in grip strength with all-cause and cardiovascular mortality in a European older population. *Clinical Medicine Insights: Cardiology*, 2018;12:1179546818771894. |

| **Comments** | **Author’s response** |
| --- | --- |
| **Major comments 5)**  It is unclear why the authors considered the use of absolute grip strength given the issue with confounding by BMI or other body composition. To me, that would be inferior to the main analyses. (Addressed as Part 1)  The same applies to the BMI cutoff where I think it would be better to use Asian specific cutoffs in the main analyses. The use of different cutoffs probably would not provide much insight since the differences reflect the varying % of fat and lean mass in Asians and Europeans. (Addressed as Part 2) | Part 1:  We understand the reviewer’s concern about the inclusion of an analysis using absolute grip strength despite our strong belief that it is highly confounded by body composition. We agree that this analysis is inferior to the main analysis that accounts for body weight, but feel it is important to include as part of our sensitivity analyses to help support and display the impact controlling for body weight has on the relationship between grip strength and T2DM. Many previous studies have also included this information either in their results (Lee et al, *PLoS One,* 2016; Chun et al, *PLoS One,* 2019; Li et al, *Front Physiol,* 2018;) or as supplementary material (Hao et al, *Front Physiol,* 2020), showing a stronger association between relative grip strength and cardiometabolic disease compared to absolute grip strength. As we point out in our discussion (page 17, line 308-310), the relationship between absolute grip strength and T2DM is weaker than when controlling for body weight, further supporting the importance of controlling for body weight when examining this association.   - Lee WJ, Peng LN, Chiou ST, et al. Relative Handgrip Strength Is a Simple Indicator of Cardiometabolic Risk among Middle-Aged and Older People: A Nationwide Population-Based Study in Taiwan. *PLoS One*. 2016;11(8):e0160876. - Chun SW, Kim W, Choi KH. Comparison between grip strength and grip strength divided by body weight in their relationship with metabolic syndrome and quality of life in the elderly. *PLoS One*. 2019;14(9):e0222040. - Li D, Guo G, Xia L, et al. Relative Handgrip Strength Is Inversely Associated with Metabolic Profile and Metabolic Disease in the General Population in China. *Front Physiol*. 2018;9:59.. - Hao G, Chen H, Ying Y, et al. The Relative Handgrip Strength and Risk of Cardiometabolic Disorders: A Prospective Study. *Front Physiol*. 2020;11:719.   Part 2:  We agree with the reviewer that body composition of the Asian population is viewed differently than that of the European descent populations. However, as is pointed out in the recommendations from the WHO expert consultation (2004) on ‘Appropriate BMI for Asian populations and its implications for policy and intervention strategies’, there is not a clear BMI cut-off point for all Asians for overweight or obesity. The risk varies from 22 kg/m^2^ to 25 kg/m^2^ in different populations and the high risk varies from 26 kg/m^2^ to 31 kg/m^2^. Thus, the WHO suggestion was that the WHO BMI cut-off points for the general population should be retained as international classifications. Due to this recommendation, we feel that using the international classification as our main analyses is most well-suited and has also been used by others (Yoon, *Lancet,* 2006; NCD Risk Factor Collaboration, *Lancet,* 2017). For the interest of the readers, and especially should future guidelines better establish or recommend the use of the Asian cut-points in analyses, we have included our sensitivity analyses using the Asian cut-points within our revised manuscript. Similar to our main analyses, we observe significantly lower odds of T2DM in both normal weight and overweight/obese individuals who are strong.     - WHO Expert Consultation. Appropriate body-mass index for Asian populations and its implications for policy and intervention strategies. Lancet. 2004;363(9403):157-163. - Yoon KH, Lee JH, Kim JW, et al. Epidemic obesity and type 2 diabetes in Asia. *Lancet*. 2006;368(9548):1681-1688. - NCD Risk Factor Collaboration. Worldwide trends in body-mass index, underweight, overweight, and obesity from 1975 to 2016: a pooled analysis of 2416 population-based measurement studies in 128·9 million children, adolescents, and adults. *Lancet*. 2017;390(10113):2627-2642. |
| **Major comments 6)**  The Discussion is a bit long and I think it is not necessary to describe in details the studies the author compared their findings with. | We thank the reviewer for their comment and have made many modifications to shorten our discussion. Due to the many changes, we have not included these in the review response document and would like to direct the reviewer to the manuscript with track changes to see our specific modifications. |
| **Minor comments 1)**  A previous genetic study suggested a causal role of grip strength in risk of type 2 diabetes and can be discussed in the manuscript (PMID: 30798333). | We appreciate this great suggestion. Thus, we have added this finding in the discussion section in the revised manuscript as shown in the sentence below (page 18, line 335-338):  “Indeed, a bi-directional Mendelian randomization study to investigate the effects of markers of grip strength on T2DM showed that higher grip strength was associated with lower T2DM risk and conversely showed that T2DM was associated with lower grip strength.” |

| **Comments** | **Author’s response** |
| --- | --- |
| **Minor comments 2)**  Line 230: The number of participants in reference 23 should be 0.42 million | We apologize for our overt error in reporting sample size. Based on the reviewer’s recommendation to reduce the details about comparison studies in the discussion, this has been removed entirely. |
| **Minor comments 3)**  Line 303-304: Stratified analyses by major confounders: This appears confusing as I think the main purpose is to use stratified analyses to explore effect modification rather than controlling for confounding? | We thank the reviewer for their attention to detail and agree that the stratified analyses is not for exploring confounding. We have removed the phrase ‘by major confounders’ (Page 19, line 359) and replaced with ‘to explore effect modification’. |
| **Minor comments 4)**  Line 309-310: Would be good to elaborate more on why there are issues with generalisability. | We have expanded on why we feel our results may have issues with generalizability within our manuscript as follows (page 19-20, line 368-371):  “Our study was limited to Korean adults who lived in Yangpyeong City. Therefore, the results of our study may only be generalized to adults living in rural areas in Korea or the Korean population as a whole.” |

**■ Responses to Reviewer #2’s comments**

| **Comments** | **Author’s response** |
| --- | --- |
| **General comments**  Thank you very much for the opportunity to review the study entitled “Associations between relative grip strength and type 2 diabetes mellitus: The Yangpyeong cohort of the Korean genome and epidemiology study”. The manuscript under review assesses whether relative grip strength is associated with type 2 diabetes mellitus (T2DM) prevalence independently of BMI among Korean elderly.  A major strength of this study is acknowledging the use of relative handgrip strength as a safe and convenient assessment for diabetes risk prediction in a representative ethnic-homogenous population. | Thank you for the encouraging comment. |

| **Comments** | **Author’s response** |
| --- | --- |
| **Major comments 1)**  In the introduction, the underlying rationale to explore the association of relative grip strength and T2DM independent of and combined with BMI is not clearly addressed by simply stating that “Previous studies investigating the relationship between grip strength and T2DM have controlled for BMI, however, few have investigated relative grip strength and T2DM independent of and combined with BMI.” Previous studies regard BMI as a potential confounder to adjust for because grip strength is known to be correlated with body size (weight, height and BMI) and BMI is a risk factor for diabetes. Can the authors elaborate why examining the association of interest independent of BMI? What is the rationale behind employing a combined association of RELATIVE GRIP STRENGTH and T2DM with BMI? | We agree this is an important consideration. There is a strong and well-known relationship between BMI and T2DM. So, BMI status may have a significant impact on the association between relative grip strength and T2DM. In previous studies, BMI was used as one of the confounding variables because the researchers were only concerned with the independent association between grip strength and T2DM. In contrast, we are investigating the impact of BMI on the association between relative grip strength and T2DM. So, we address the questions of whether the association was independent of BMI, and whether T2DM prevalence differs between the different combinations of relative grip strength and BMI. To provide a better explanation, we corrected the sentence in the introduction section in the revised manuscript as shown in the sentence below (page 4-5, line 44-62).  “Studies have shown that relative grip strength, which is grip strength divided by body weight or BMI, may be more strongly associated with metabolic syndrome and cardiometabolic risk factors (e.g., blood pressure, triglyceride, fasting glucose) than absolute grip strength. Thus, relative grip strength may have a stronger association with T2DM than absolute grip strength.  Obesity is attributed to insulin resistance by permanently increasing free fatty acids in plasma and reducing glucose uptake by muscle. Many previous studies have elucidated the importance of obesity, assessed by body mass index (BMI) ≥30 kg/m^2^, as a prominent risk factor for T2DM. Thus, the association between grip strength and T2DM may depend on BMI status. Previous studies investigating the relationship between grip strength and T2DM have controlled for BMI [18], however, few have investigated how the association between relative grip strength and T2DM is varied with BMI status.  It is important to examine the combined effects of muscular strength and overweight/obesity since both are important to develop public health recommendations and policies. For example, if people with overweight/obesity can reduce the risk of T2DM by improving muscular strength, this has a large clinical and public health impact since overweight/obesity is prevalent in most mid- and high-income countries [19]. Further, there is still limited data on the relative contributions of muscular strength vs. overweight/obesity to T2DM.” |

| **Comments** | **Author’s response** |
| --- | --- |
| **Major comments 2)**  In the logistic regression model 3, the authors controlled for BMI along with other potential confounders but hastily concluded that the association of interest was independent of BMI. These points need clarification along with solid evidence. Other strategies such as automatic variable selection can be used to support or refute whether adjustment for BMI is required (1). In addition, in line 244-246 "however, the association between grip strength and T2DM remained even after controlling for BMI. This suggests that there is a significant relationship between muscle strength and T2DM independent of BMI.", the sentence lacks reasoning. I think the association remain significant after adjustment for a third variable is not enough to say the relationship is independent of the third variable. | We thank the reviewer for the feedback. We hope our further discussion here more clearly explains to support our statement and conclusion.  In many earlier studies (Lee et al, *Circulation,* 2011), including some of our own (Ingle et al, *Heart*. 2017; Goel et al, *Am Heart J*. 2011; Kwakernaak et al, *J Am Soc Nephrol,* 2013), similar statistical analyses and interpretations have been utilized. For example, in the study by Ingle L, et al. (2017) examining the association between cardiorespiratory fitness and metabolic syndrome, they show that there was a strong association between cardiorespiratory fitness and metabolic syndrome in British men. After controlling for BMI in their models the association remains significant. Therefore, they conclude that the association was independent of BMI.  By including BMI in our model, we are aiming to control for its effect on both the exposure (handgrip strength) and outcome (T2DM). When the addition of the covariate (i.e. BMI) eliminates the relationship between exposure and outcome, we would suggest that this covariate (i.e. BMI) largely explains the relationship between our exposure and outcome. However, when we control for the covariate and our significant relationship between exposure and outcome remains, this would suggest that our exposure and outcome are still related even after controlling for a covariate (i.e. BMI). After adding BMI to the model, we still observe a significant relationship between relative handgrip strength and T2DM (Table 2). Thus, we feel this suggests this relationship withstands the adjustment for BMI and may therefore be independent of BMI. However, in accordance with the reviewer, we understand that statistically controlling for a confounder is not always the strongest approach to definitively confirm the independence of a relationship.  We feel the independence of BMI from our exposure/outcome relationship is also evident when we look at our joint analyses (Fig 2). In this analysis, we see that greater relative grip strength is associated with lower odds of T2DM for BOTH the normal weight and the overweight/obese groups. Additionally, we did not observe any significant interaction effects for normal weight and overweight/obesity groups (p=0.673). Thus, regardless of BMI, having a stronger absolute grip strength is associated with lower odds of T2DM. We again feel that because the beneficial relationship between strength and T2DM is observed in both BMI groups, this would suggest our observed relationship is independent of BMI. Similar to the analyses above, prior studies have also used joint analyses to examine the independence of relationships between exposures and outcomes. For example, Kwakernaak AJ, et al. (2013) report a significant association between waist-hip-ratio and filtration fraction of renal, independent of BMI, using joint analysis.  In combination, we feel our two analyses (adjustment for BMI in Table 2 as well as joint stratification in Figure 2) support the conclusion of a relationship between relative handgrip strength and T2DM independent of BMI.   - Lee DC, Sui X, Artero EG, et al. Long-term effects of changes in cardiorespiratory fitness and body mass index on all-cause and cardiovascular disease mortality in men: the Aerobics Center Longitudinal Study. *Circulation*. 2011;124(23):2483-2490. - Ingle L, Mellis M, Brodie D, et al. Associations between cardiorespiratory fitness and the metabolic syndrome in British men. *Heart*. 2017;103(7):524-528. - Goel K, Thomas RJ, Squires RW, et al. Combined effect of cardiorespiratory fitness and adiposity on mortality in patients with coronary artery disease. *Am Heart J*. 2011;161(3):590-597. - Kwakernaak AJ, Zelle DM, Bakker SJ, et al. Central body fat distribution associates with unfavorable renal hemodynamics independent of body mass index. *J Am Soc Nephrol*. 2013;24(6):987-994. |
| **Major comments 3)**  In line 43, please be more specific in the sentence “may be more strongly associated with various diseases” as of what diseases were RELATIVE GRIP STRENGTH associated with? Are these diseases also correlated to the outcome of interest, which is necessary to address? | We thank the reviewer for their attention to detail. We have modified this sentence to be more specific, as shown below and included in the revised manuscript (page 4, lines 43-44):  “Studies have shown that relative grip strength, which is grip strength divided by body weight or BMI, may be more strongly associated with cardiometabolic risk factors (e.g., blood pressure, triglyceride, fasting glucose) than absolute grip strength [12-14]” |

| **Comments** | **Author’s response** |
| --- | --- |
| **Major comments 4)**  Lack of justification for the underlying rationale subsequently leads to a few arguable points in the methods and statistical analysis part. In the logistic regression analysis to estimate the odds ratios (OR) of T2DM with the relative grip strength quintiles, important confounders such as socioeconomic position, nutritional intake were not controlled because they were not available. Additionally, is there a need to adjust for “living status” in Model 2?  Also, I wonder if glycaemic status is another important confounder to consider within the association of interest. As suggested in the reference 12, “relative HGS has stronger associations with metabolic syndrome and its component parameters, including high-density lipoprotein cholesterol (HDLC), TG, fasting glucose, and blood pressure”.  Without considering important confounders can lead to confounding bias while accounting for a non-confounder may introduce spurious associations within the association. Both affect the validity of the study that mask the true association and lead to incorrect results and interpretation. However, even after adjusting for potential confounders, residual confounding remains. | We agree with the reviewer that confounders in a statistical model should be considered very carefully as to reduce the likelihood of confounding bias and/or spurious associations. The selection of our models was therefore based on previous literature and scientific evidence.  As pointed out in our limitations, we are unable to address the potential confounding from socioeconomic status or nutritional intake since the data were not available. In regard to ‘living status’, previous studies report that living alone is associated with increased diabetes risk (Lidfeldt et al. *Diabetes Care*, 2005; Fink et al., *PloS one*, 2017; Meisinger et al., *Psychosomatic Medicine*, 2009). Further, this significant association has also been recently reported specifically in Korean adults (Nam GE et al. *Scientific Reports*, 2021). Based on this prior literature, we felt it was important to include “living status” as a confounder in our analysis. However, in lieu of your comment, we have also conducted our analyses not controlling for ‘living status’. In Model 3 without living status, we observed ORs (95% CIs) for the second, third, fourth, and fifth quintiles of relative grip strength were 0.73 (0.53-1.02), 0.68 (0.48-0.96), 0.71 (0.50-1.02) and 0.48 (0.31-0.74), respectively. These results are very similar to those reported in our main analyses. Although the removal of living status would not dramatically alter what is being reported, we feel the prior literature supports its inclusion.    In regard to the inclusion of a confounder related to glycaemic status, we agree with the reviewer that there is a significant association between grip strength and fasting glucose. However, fasting glucose was used as a diagnostic value for the determination of T2DM in the present manuscript. High fasting glucose (≥ 126 mg/dL) was categorized as T2DM. Thus, we believe that adjusting for fasting glucose is not necessary. In addition, in a similar previous study that examined the association between grip strength and incidence of T2DM, there was no significant interaction effect on glycaemic status (normal or impaired fasting glucose) (Wang Y et al, *Mayo Clinic Proceedings,* 2019). Therefore, we did not include glycemic status as a confounder in our model.   - Lidfeldt J, Nerbrand C, Samsioe G, et al. Women living alone have an increased risk to develop diabetes, which is explained mainly by lifestyle factors. *Diabetes Care*, 2005;28(10):2531-2536. - Fink A, Buchmann N, Tegeler, C, et al. Physical activity and cohabitation status moderate the link between diabetes mellitus and cognitive performance in a community-dwelling elderly population in Germany. *PloS one*, 2017;12(10):e0187119. - Meisinger C, Kandler U, Ladwig KH. Living alone is associated with an increased risk of type 2 diabetes mellitus in men but not women from the general population: the MONICA/KORA Augsburg Cohort Study. *Psychosomatic Medicine*, 2009;71(7):784-788. - Nam GE, Kim W, Han K, et al. Association between living alone and incident type 2 diabetes among middle-aged individuals in Korea: a nationwide cohort study. *Scientific Reports*, 2021;11(1):1-8. - Wang Y, Lee DC, Brellenthin AG, et al. Association of muscular strength and incidence of type 2 diabetes. *Mayo Clinic Proceedings,* 2019;94(4):643-651. |
| **Major comments 5)**  In Figure 1, why choose 70 as a cut-off age in stratification analysis? The >70 age group has a small sample size, especially among the strongest RELATIVE GRIP STRENGTH quintile, which may lack statistical power to detect the strength of associations.  Also, it would be good to report the sex-specific difference in the association between RELATIVE GRIP STRENGTH and T2DM, like the strength of association in female was not significant as in male. | - We thank the reviewer for their suggestion and highlighting the disparity in sample size for our age-specific stratification. We have re-examined the cut-off value of age and re-stratified based on the age of 65 years. We chose to use 65 years of age not only because it creates a more even sample size distribution, but because it is also used scientifically to define the elderly. Please see our updated results in Fig 1. You will now see that the relationship between relative grip strength and T2DM is quite similar in both age-strata. Secondly, we have added a paragraph on the gender difference in association in the discussion section in the revised manuscript (page 18-19, line 342-355), as shown in the sentence below:   “We performed a subgroup analysis to determine whether the association between relative grip strength and T2DM varies according to each confounder. Although there were no significant interaction effects in all strata, all subgroups showed some differences in association according to classification, especially when stratified by sex, there was a significant trend only in men. These differences are difficult to explain clearly. One possible explanation is that the different glycaemic profiles between men and women. Impaired fasting glucose more common in men than women, and impaired glucose tolerance are more common in women [51, 52]. So, differences in the association between men and women may be affected by the diagnostic criteria for T2DM used in our study. However, further studies, especially prospective studies, with larger samples are clearly warranted to examine the sex-specific difference relationship between relative grip strength and T2DM.” |

| **Comments** | **Author’s response** |
| --- | --- |
| **Major comments 6)**  In line 195, “Fig 2 demonstrates the additive effects of combining BMI and relative grip strength on the odds of T2DM.” What do you mean by "additive effects"? The analysis in Figure 2 seems to examine the statistical interaction of BMI on RELATIVE GRIP STRENGTH by stratification among subgroups. Is this an appropriate analysis? I doubt about it to treat BMI as an effect modifier as from the Figure 1 results, it is not certain to claim the association between RELATIVE GRIP STRENGTH and T2DM is independent of BMI. I might have missed this, but what do the stratum-specific ORs by BMI look like? | We apologize to the reviewer for the confusion. We have modified the phrase ‘additive effects of combining’ to ‘combined association between’ (page 13, line 213).  Additionally, as requested by Reviewer 1, we have performed formal interaction analyses to supplement our analysis in Fig 2. As mentioned above and now in text, we observed the stratum specific ORs by confounders. The stratum-specific ORs by BMI were presented in Supplementary Table 3. Within strata, relative handgrip strength was only associated with the odds of T2DM in obese individuals. Thus, we chose to examine whether T2DM prevalence differs among the various combinations of relative grip strength and BMI in Fig 2. Our analytical procedure replicates previous studies who were also investigating the independent and combined associations between variables of interest (Lee et al, *Circulation,* 2011; Weinstein et al. *Jama*, 2004; Goel et al. *American heart journal*, 201121392616).   - Lee DC, Sui X, Artero EG, et al. Long-term effects of changes in cardiorespiratory fitness and body mass index on all-cause and cardiovascular disease mortality in men: the Aerobics Center Longitudinal Study. *Circulation*, 2011;124(23):2483-2490. - Weinstein AR, Sesso HD, Lee IM. Relationship of physical activity vs body mass index with type 2 diabetes in women. *Jama*, 2004;292(10):1188-1194. - Goel K, Thomas RJ, Squires, RW, et al. Combined effect of cardiorespiratory fitness and adiposity on mortality in patients with coronary artery disease. *American heart journal*, 2011;161(3):590-597. |
| **Major comments 7)**  Information bias is another issue not addressed in the discussion. This can occur in measuring and classifying covariates, exposure and outcomes and may lead to misclassification. For example, “Do you regularly participate in exercise that elicits sweat” is a qualitative question with a "Yes/No" answer to define “regular exercise”. Furthermore, inaccuracy in classifying self-reported lifestyle variables on smoking, alcohol drinking status, and medical conditions (e.g. hypertension, dyslipidemia, T2DM) can exist. Social desirability bias, as a form of information bias, cannot be ruled out here. | We thank the reviewer for their comment and agree these are important considerations. We have included these as limitations in the Discussion section, as shown below and in the revised manuscript (page 20, line 372-378):  “Finally, many confounders were collected through a self-report questionnaire thus introducing the potential for information bias and bias due to social desirability, which could subsequently lead to misclassification. This potential bias should be considered when interpreting the results from this study. However, we tried to potentially counter the limitation by setting that the main exposure was measured and that T2DM was determined not only based on self-report of the disease, but also medications and fasting glucose.” |
| **Major comments 8)**  In the discussion, the authors acknowledge that it is not possible to establish a causal relationship in this cross-sectional study and stated that T2DM can induce loss of muscle mass and muscle strength in line 299-301. Therefore, reverse causality bias is possible (2). | Following this comment, we have rephrased the limitations of our study in the Discussion section, as shown below in the revised manuscript (page 19, line 362-365):  “First, this study is cross-sectional in design, therefore it is not possible to establish a causal relationship between relative grip strength and the prevalence of T2DM, and reverse causality (e.g., T2DM could induce the loss of muscle mass and strength) is possible.” |

| **Comments** | **Author’s response** | |
| --- | --- | --- |
| **Major comments 9)**  A complete case analysis is based on the assumption that variables were missing completely at random. This may not be valid sometimes if the missing data is dependent on a variable affecting both T2DM risks and missingness of data. Is there a valid assumption in this study to utilize complete case analysis? To validate the assumption, are there sensitivity analyses using different methods such1 as inverse probability weight to simulate the missing data? | We appreciate the reviewers comment and are happy to explain why we chose to use a complete case analysis instead of imputation. We would first like to acknowledge that this approach does increase the risk of bias due to the reduction in sample size. However, Hair et al. (2019) reported that if the extent of missing data less than 10% of the sample, the result of the analysis is likely not to be affected by using a complete case analysis approach. (Hair JF et al. Multivariate data analysis, 8th edition. 2019). In our dataset, among a total of 3,351 participants, 332 participants were excluded for reasons such as non-compliance with ≥ 8 hours of fasting and a history of cancer/stroke/heart disease. Of 3,019 participants, there were 39 participants (1.3%) missing at least one covariate, and 179 (5.9%) participants with no report of our main exposure, grip strength. The small number of missing values in our sample would suggest, based on Hair et al, that the risk of bias due to case deletion would be small and have minimal influence on our results. Therefore, we feel comfortable in using a complete case analysis approach in this study. However, there are still concerns about bias. So, we performed a sensitivity analysis using the mean substitution method for grip strength to examine how different the results depend on the missing values. As a result of the analysis, the association was weakened but still showed a similar trend to the results of our study using a complete case approach. The results are presented in the table below. **Table 2. Odd Ratios of T2DM by quintiles of relative grip strength.** \| \| \| \| \|; **Relative grip strength** \| **N** \| **Cases** \| **OR (95% CI)** \| \|; \| \| \| **Model 1** \| **Model 2** \| **Model 3**; Q1 (weakest) \| 604 \| 108 \| 1.00 (reference) \| 1.00 (reference) \| 1.00 (reference); Q2 \| 604 \| 96 \| 0.90 (0.67-1.22) \| 0.88 (0.64-1.20) \| 0.90 (0.66-1.23); Q3 \| 604 \| 82 \| 0.77 (0.56-1.05) \| 0.74 (0.53-1.02) \| 0.77 (0.55-1.07); Q4 \| 604 \| 67 \| **0.63 (0.45-0.88)** \| **0.63 (0.44-0.88)** \| **0.67 (0.47-0.96)**; Q5 (strongest) \| 603 \| 53 \| **0.50 (0.35-0.71)** \| **0.54 (0.37-0.78)** \| **0.60 (0.40-0.90)**; P for linear trend \| \| \| **<.001** \| **<.001** \| **0.005**; Per SD in relative grip strength^a^ \| \| \| **0.75 (0.65-0.86)** \| **0.77 (0.66-0.88)** \| **0.81 (0.69-0.94)**; ^a^A 1 SD in relative grip strength is equivalent to 0.11kg. Model 1 was adjusted for sex and age (years). Model 2 was adjusted for Model 1 plus smoking status (never, former, current), current alcohol drinking status (yes or no), regular exercise (yes or no), living with family (yes or no), ≥high school graduate (yes or no), family history of diabetes (yes or no), hypertension (yes or no), dyslipidemia (yes or no). Model 3 was adjusted for Model 2 plus body mass index (kg/m^2^). \| \| \| \| \| | |
| **Comments** | | **Author’s response** |
| **Major comments 10)**  For generalizability, the sentence in line 310-311, “the participants were limited to only one ethnic group and living only in one area, so it is difficult to generalize our findings to the population” needs clarification. What does “the population” refer to? | | Thanks for your important comment. As also mentioned by Reviewer 1, we have modified our sentence in the revised manuscript and included below: (page 19-20, line 367-371).  “Second, our study was limited to Korean adults who lived in Yangpyeong City. Therefore, the results of our study may only be generalizable to adults living in rural areas in Korea or the Korean population as a whole.” |
| **Major comments 11)**  Please provide the results of sensitivity analyses that assess the robustness of statistical models in detail if possible, to substantiate the findings. | | We thank the reviewer for their comment. We have now added a Supplementary file to our manuscript to further expand on our sensitivity analyses that are not included within the manuscript text. Within this file, you can find the results of analyses of 1) both tertiles and quartiles of relative grip strength (Table 1), 2) the odds of T2DM by quintile of relative grip strength when using BMI instead of body weight (Table 2), and 3) subgroup analyses such as by BMI category (Table 3). |
| **Minor comments 1)**  In line 167, “The addition of BMI in Model 3..”, please be clearer in the description to additionally adjust for BMI based on Model 2 confounders. | | We revised the sentence more clearly following the sentence below (page 12, line 197-199).  “In Model 3, however, the addition of BMI weakened the association between relative grip strength and T2DM although a significant linear trend was still observed (p=0.002).” |

| **Comments** | **Author’s response** |
| --- | --- |
| **Minor comments 2)**  For the study population, a reference to the cohort profile of the study population would be helpful when discussing generalizability. | We have added a reference for the cohort profile in the Materials and Methods section in the revised manuscript (page 5, line 67) (Kim et al, *Int J Epidemiol*. 2017).   - Kim Y, Han BG; KoGES group. Cohort Profile: The Korean Genome and Epidemiology Study (KoGES) Consortium. *Int J Epidemiol*. 2017;46(2):e20. |
| **Minor comments 3)**  The subsection "clinical examination" under the "Material and Methods" section describes not only clinical examination. "covariates" would be a better word. | We thank the reviewer for their comment and have changed this heading from ‘Clinical Examination’ to ‘Covariates’ (page 7, line 111). |
| **Minor comments 4)**  Fig2. The 3D graph does not add values to the visual aids but may be quite confusing. A simple 2-by-2 table would be good for results presentation. | We thank the reviewer for bringing to our attention the difficulty some readers may have when looking at a 3D graph. Although this data could be presented in table form, we would like to have a visual representation of the magnitude of the odds ratios for the readers to see. To compromise, we have changed the style of Fig 2 as presented below. To no longer be in 3D format but still visually show the joint association between relative handgrip strength and body mass index. |

Odds ratios (95% confidence intervals) of type 2 diabetes mellitus

|  |  |  |
| --- | --- | --- |
| weak and overweight/obese | 1 | 1 |
| weak and normal weight | 0.68 | 1 |
| strong and overweight/obese | 0.65 | 1 |
| strong and normal weight | 0.49 | 1 |

| **Comments** | **Author’s response** |
| --- | --- |
| **Minor comments 5)**  Causal language was used sometimes within the text. For example, in line 195 when describing Figure 2, “…the additive effects of combining BMI and relative grip strength…” and line 269, the word “effects” would convey causal connotation. | We appreciate this important comment. We modified the phrase ‘additive effects of combining’ to ‘combined association between’ (Page 13, line 225).  We double-checked, but we did not use the word “effects” in line 269. However, we checked other places and made sure that we did not use “effects” in interpreting the results.  We thought that you have pointed out the ‘effect’ in the following sentence (page 15, line 268). “One possible explanation of the different findings between ours and earlier studies is that both prior studies used absolute grip strength without considering the strong effect of body weight on grip strength.”  Thus, we revised the sentence following the sentence below (page 11, line 182-184).  “One possible explanation of the contradicting findings may be that both prior studies used absolute grip strength without considering the strong association between body weight and grip strength.” |
| **Minor comments 6)**  Please be consistent when referring to relative grip strength or absolute grip strength throughout the manuscript to avoid confusion. For instance, (line 242-247) “In our study, however, the association between grip strength and T2DM remained even after controlling for BMI.” | We appreciate the reviewers comment and feel this improves the readability of our manuscript. Throughout the manuscript, we have ensured that grip strength is always prefaced with either ‘relative’ or ‘absolute’ to reduce any confusion readers may have. Given this occurred in several instances, we have not included each specific page and line here in the reviewer response document, but hope the reviewer can see our effort throughout when reviewing the tracked changes document. |
