# Supplementary material for: Associations between relative grip strength and type 2 diabetes mellitus: The Yangpyeong cohort of the Korean genome and epidemiology study
Source: PLoS One. 2021 Aug 26;16(8):e0256550. doi: 10.1371/journal.pone.0256550 (PMC8389482; doi:10.1371/journal.pone.0256550)
Supplement: Attachment — Submitted filename: Response to Reviewers.docx [file pone.0256550.s005.docx]

**Associations between Relative Grip Strength and Type 2 Diabetes Mellitus:**

**The Yangpyeong Cohort of the Korean Genome and Epidemiology Study**

We thank the reviewer for the comments and are grateful for the opportunity to provide a revised version for consideration. We responded to the feedback below with direct locations (page and line numbers) of changes in the revised manuscript by using track changes.

**■ Responses to Editors comments**

| **Comments** | **Author’s response** |
| --- | --- |
| **Journal Requirements:**  1) Please review your reference list to ensure that it is complete and correct. If you have cited papers that have been retracted, please include the rationale for doing so in the manuscript text, or remove these references and replace them with relevant current references. Any changes to the reference list should be mentioned in the rebuttal letter that accompanies your revised manuscript. If you need to cite a retracted article, indicate the article’s retracted status in the References list and also include a citation and full reference for the retraction notice. | We have rechecked all references and identified that reference 1 has been retracted (World Health Organization. Global report on diabetes. 2016). We have replaced this reference with the following:   - Saeedi P, Salpea P, Karuranga S, Petersohn I, Malanda B, Gregg EW, et al. Mortality attributable to diabetes in 20–79 years old adults, 2019 estimates: Results from the International Diabetes Federation Diabetes Atlas*. Diabetes research and clinical practice*. 2020;162:108086. |

**■ Responses to Reviewer #1’s comments**

| **Comments** | **Author’s response** |
| --- | --- |
| **Minor comments 1)**  I think it would deserve more description to explain possible differences in association when BMI was used to normalize grip strength instead of body weight alone, where the associations were attenuated (in terms of effect size) when BMI was used. | We thank the reviewer for the important suggestion and have included a discussion of this topic. As pointed out, the associations were attenuated when using BMI-normalized grip strength (Supplementary Table 2) compared to body weight-normalized grip strength on diabetes. It is important to recognize that in Model 3 BMI was further adjusted because it’s important to control for the effect of BMI on our outcome, diabetes. This further adjustment may partially explain the attenuated results, however, the odd ratio per 1 SD is still significant (Odd ratio: 0.79; 95% Confidence interval: 0.65-0.97) in Model 3 in Supplementary Table 2.  We are not currently aware of any literature comparing weight-normalized vs. BMI-normalized grip strength on diabetes. A previous study has reported that weight-normalized grip strength has a greater association with metabolic syndrome than BMI-normalized grip strength (Chun SW et al, *PloS one*, 2019), similar to our findings although the study outcome is different. Therefore, further studies are warranted to confirm our findings on diabetes.   - Chun SW, Kim W, Choi KH. Comparison between grip strength and grip strength divided by body weight in their relationship with metabolic syndrome and quality of life in the elderly. *PloS one*, 2019;14(9):e0222040.. doi: 10.1371/journal.pone.0222040.   Following the comment, we have added the following sentences to our revised manuscript (page 17, line 327-335).  "However, there was a difference in the results of analysis when using the relative grip strength normalized by body weight or BMI. In our results, the associations were attenuated when using BMI-normalized grip strength (Supplementary Table 2) compared to body weight-normalized grip strength on the prevalence of T2DM. One previous study has reported that weight-normalized grip strength has a greater association with metabolic syndrome than BMI-normalized grip strength [12]. There is no study comparing weight and BMI normalized grip strength on the prevalence of T2DM. Therefore, further studies are clearly warranted to examine the difference between relative grip strength normalized by body weight or BMI.” |

| **Comments** | **Author’s response** |
| --- | --- |
| **Minor comments 2)**  Regarding generalizability, I think the key thing is whether studies in Koreans can be applied to other populations, such as Chinese. | We agree and understand the reviewer’s concern. We have rephrased the limitations of our study in the Discussion section of the revised manuscript (page 19, line 373-375), as shown below:  “Therefore, the results of our study may only be generalizable to adults living in rural areas in Korea or other Asian populations, especially Northeast Asians such as Chinese or Japanese people [53].”  We have additionally included the reference below that the genetic polymorphisms related to type 2 diabetes were not ethnically different between Korean, Japanese, and Chinese.   - Takeuchi M, Okamoto K, Takagi T, Ishii H. Ethnic difference in patients with type 2 diabetes mellitus in inter‐East Asian populations: a systematic review and meta‐analysis focusing on gene polymorphism. *Journal of diabetes*. 2009;*1*(4):255-262. doi: 10.1111/j.1753-0407.2009.00040.x |
